# Regional Brain Differences in Cortical Thickness, Surface Area and Subcortical Volume in Individuals with Williams Syndrome

**DOI:** 10.1371/journal.pone.0031913

**Published:** 2012-02-15

**Authors:** Shashwath A. Meda, Jennifer R. Pryweller, Tricia A. Thornton-Wells

**Affiliations:** 1 Department of Molecular Physiology and Biophysics, Center for Human Genetics and Research, Vanderbilt University, Nashville, Tennessee, United States of America; 2 Interdisciplinary Studies in Neuroimaging of Neurodevelopmental Disorders, The Graduate School, Vanderbilt University, Nashville, Tennessee, United States of America; Beijing Normal University, Beijing, China

## Abstract

Williams syndrome (WS) is a rare genetic neurodevelopmental disorder characterized by increased non-social anxiety, sensitivity to sounds and hypersociability. Previous studies have reported contradictory findings with regard to regional brain variation in WS, relying on only one type of morphological measure (usually volume) in each study. The present study aims to contribute to this body of literature and perhaps elucidate some of these discrepancies by examining concurrent measures of cortical thickness, surface area and subcortical volume between WS subjects and typically-developing (TD) controls. High resolution MRI scans were obtained on 31 WS subjects and 50 typically developing control subjects. We derived quantitative regional estimates of cortical thickness, cortical surface area, and subcortical volume using FreeSurfer software. We evaluated between-group ROI differences while controlling for total intracranial volume. In post-hoc exploratory analyses within the WS group, we tested for correlations between regional brain variation and Beck Anxiety Inventory scores. Consistent with our hypothesis, we detected complex patterns of between-group cortical variation, which included lower surface area in combination with greater thickness in the following cortical regions: post central gyrus, cuneus, lateral orbitofrontal cortex and lingual gyrus. Additional cortical regions showed between-group differences in one (but not both) morphological measures. Subcortical volume was lower in the basal ganglia and the hippocampus in WS versus TD controls. Exploratory correlations revealed that anxiety scores were negatively correlated with gray matter surface area in insula, OFC, rostral middle frontal, superior temporal and lingual gyrus. Our results were consistent with previous reports showing structural alterations in regions supporting the socio-affective and visuospatial impairments in WS. However, we also were able to effectively capture novel and complex patterns of cortical differences using both surface area and thickness. In addition, correlation results implicate specific brain regions in levels of anxiety in WS, consistent with previous reports investigating general anxiety disorders in the general population.

## Introduction

Williams syndrome (WS) is a rare genetic neurodevelopmental disorder caused by a microdeletion of approximately 25 genes on chromosome 7q11.23. WS is associated with a unique behavioral and cognitive profile, which involves mild to moderate intellectual disability. Individuals with WS often demonstrate increased non-social anxiety and phobias, paired with hypersociability and heightened empathy. In social interactions, persons with WS are often impulsive, lack social inhibition and have a lack of fear of strangers [1], [2], [3]. The WS cognitive profile is characterized by deficits in visuospatial, motor and visuomotor abilities and relative strengths in face and object recognition, expressive language and music processing skills [3], [4], [5], [6], [7].

Over the past 10–15 years, several structural and functional neuroimaging studies have characterized brain anatomical differences in WS and have identified putative neural correlates for specific aspects of the WS phenotype. Post-mortem and *in vivo* structural studies have shown a global reduction in brain volume [8], [9], [10], [11]. The literature also describes a higher ratio of frontal to posterior cerebral volume in WS compared to typically developing (TD) controls [8]. Previous magnetic resonance imaging (MRI) studies that employed automated methods, such as voxel-, tensor- or deformation-based morphometry, consistently showed reduced grey matter volume in the intraparietal sulcus, occipitoparietal sulcus, brain stem and occipital lobe regions in WS versus TD controls [12], [13], [14], [15]. Other regional differences, such as in the orbitofrontal cortex (OFC), superior temporal gyrus, insula and the anterior cingulate cortex are often reported inconsistently across studies, with some studies reporting greater grey matter volume and others reporting less or no differences [9], [10], [12], [13], [14], [15]. More recent studies have used surface- and mesh-based analyses to measure sulcal and gyral pattern differences in WS. Gaser et al. [16] used an automated measure of gyrification and found greater gyrification in the cuneus, occipital and medial frontal lobes in the WS group versus TD controls. A surface-based analysis by Van Essen et al. [17] reported reductions in sulcal depth in the cingulate, frontal operculum, and anterior and posterior intraparietal regions, along with increases in sulcal depth in the hippocampus, cuneus, angular gyrus, superior temporal gyrus, medial frontal gyri, and parieto-occipital regions.

Several studies have now reported using diffusion tensor imaging (DTI) to detect differences in white matter (WM) structure and connectivity. Marenco and colleagues [18] analyzed a unique subset of high functioning WS individuals and found significant differences in fiber orientation underlying the posterior regions of the brain. Hoeft et al. [19] investigated the integrity of the superior and inferior longitudinal fasciculi that connect the fronto-parietal and the temporo-parietal brain systems respectively and found increased fractional anisotropy in WS relative to normal individuals. Furthermore, this increase in anisotropy was associated with visuo-spatial deficits in WS [19]. Arlinghaus and colleagues [20] used tract-based DTI in WS and found decreased structural connectivity between regions previously reported to have morphometric differences in the WS brain, primarily in posterior regions. Most recently, Avery et al. [21] reported alterations in the structural integrity of the prefrontal-amydala white matter pathway might underlie the altered emotional reactivity and the heightened non-social fear and anxiety observed in WS.

Although a substantial body of literature exists to characterize anatomical brain differences in WS, few studies have made simultaneous, within-subject measurement of distinct morphological traits. Discrepant or lack of significant findings may be due to the likelihood that the brain volume phenotype is a combination of at least two genetically and developmentally independent traits: cortical surface area and cortical thickness [22], [23]. In order to disentangle this potential confound, we made concurrent regional cortical measurements of surface area and thickness. However, for subcortical regions, we instead used brain volume as the most appropriate measure. We hypothesized that concurrent measures of surface area and thickness would increase the sensitivity and specificity of cortical morphometric measurement and better characterize complex patterns of structural brain differences in WS, informing how those findings relate to particular aspects of the WS cognitive and behavioral phenotype [22].

Individuals with WS have preserved face and object recognition but impaired visuospatial processing, implicating a specific impairment in the dorsal (versus ventral) stream of processing. Reports from high resolution structural imaging studies, as well as from functional MRI (fMRI) studies have confirmed the anatomical specificity of these findings [14], [24]. Moreover, structural studies have also found regional alterations in brain regions governing visuospatial functioning in WS. Reiss et al. [15] used an automated voxel based morphometry method to identify decreases in gray matter volume in several areas including the superior parietal, cuneus and the middle occipital gyrus in individuals with WS. Also, the prior mentioned DTI study by Arlinghaus and colleagues [20] provides support for poor white matter integrity in tracts connecting visuo-spatial regions in WS. Thus, we expected to find cortical differences in areas related to visuospatial processing and visuomotor abilities, such as the cuneus, superior parietal, inferior parietal, premotor and other occipital regions.

Many individuals with WS have increased auditory sensitivities, heightened empathy, and with an increased emotional response and interest in music [25], [26], [27], [28]). MRI studies have implicated functional differences in the auditory cortex as well as emotional brain regions, such as the amygdala, insula and posterior cingulate [29], [30]. One study also found evidence for altered auditory processing that might involve cross-modal sensory connections between auditory (temporal) and visual (occipital) cortices [30]. Morphometric studies have also implicated increased GM volume in the superior temporal gyrus and left planum temporale with the WS musical phenotype [10], [31]. Given the above evidence, we hypothesized that we would observe morphological differences in temporal and limbic lobe regions related to the unique behavioral phenotype of WS.

Several fMRI studies have looked at WS from a social cognition perspective [32], [33], [34]. One study found that WS subjects had increased activation in the amygdala in response to threatening scenes but a blunted response to threatening faces and concluded that abnormal interactions between the OFC and amygdala are a possible neural basis for dysregulated social behavior in WS [32]. Supporting this particular functional finding, a recent DTI study found reduced integrity of WM tracts between the OFC and amygdala in WS versus TD controls [21]. In the current study, we further explored this putative neural correlate of anxiety and tested for correlations between morphometric measures and scores on the Beck Anxiety Inventory. Based on prior research, we predicted that anxiety scores would be significantly correlated with structural morphological measures in the frontal-amygdala circuit, anterior cingulate, OFC and insula [33], [35], [36]. We expected to detect specific structural alterations in additional regions governing emotional regulation and social cognition, such as the amygdala, OFC and medial frontal gyrus.

Many individuals with WS are impulsive and qualify as having attention deficit disorder. In a recent study by Mobbs et al. [5], subjects with WS demonstrated poor response inhibition in a Go/NoGo task and showed gross hypofunction of the basal ganglia system relative to TD subjects. Other behavioral studies have documented visuomotor deficits that support anecdotal reports of difficulty with balance, coordination and gait [6], [7]. Given these findings, we further hypothesized there would be differences in the basal ganglia circuit.

## Materials and Methods

The current study included 31 WS subjects (20 males; mean age 26.3) and 50 TD control subjects (27 males; mean age 28). WS participants were recruited through the annual Academy of Country Music Lifting Lives Music Camp, which is organized by the Vanderbilt Kennedy Center. All WS participants exhibited the physical, cognitive and behavioral profile of WS and previously had received a clinical diagnosis of WS and confirmatory genetic testing. TD controls were recruited using flyers and website postings with IRB-approved language targeting healthy individuals from the general population. Although many WS subjects are literate, to allow for consistent administration, regardless of reading level, the Beck Anxiety Inventory was administered to each WS participant by reading the items out loud and asking for a verbal response, which was then recorded by the trained interviewer. The two groups were tested for differences in age and sex, using an independent samples t-test and a chi-square test, respectively. Demographic characteristics along with their corresponding statistical values are detailed in Table 1.

**Table 1:** Sample summary statistics.

	TD (N = 50)	WS (N = 31)	Group Contrasts	
	Mean (SD)	Mean (SD)	t	p-value
**Age (years)**	28.0 (9.0)	26.3 (7.7)	0.41	0.74
**Intracranial Volume (Z norm)**	0.46 (0.84)	−0.75 (0.74)	6.6	4.20E-09
**Beck Anxiety Inventory**	-	6.5 (6.2)	-	-

	N (%)	N (%)	Chi-square	p-value
**Male Sex**	27 (54)	20 (64)	0.35	0.24

Group means and standard deviations for age, total intracranial brain volume estimates, and Beck Anxiety Inventory scores (in WS only), along with test statistics for between-group analyses are provided. Proportion of males is also reported by group.

High resolution 3D T1-weighted MRI scans were obtained on a 3T Philips Achieva scanner housed at the Vanderbilt University Institute of Imaging Science (Nashville, TN) with the following parameters: field of view = 256×256 mm^2^; in plane voxel resolution = 1×1 mm^2^; TR = 8.9 ms; TE = 4.6 ms; flip angle = 8°; slice thickness = 1 mm and 170 slices with no slice gap. TD control subjects and caregivers of individuals with WS gave informed consent, while participants with WS gave assent. The study was approved by the Vanderbilt University Institutional Review Board. We used an automated, non-biased atlas-based Bayesian segmentation procedure, applied in Freesurfer v.5.0 (http://surfer.nmr.mgh.harvard.edu/), to derive quantitative estimates of brain structure and to label cortical and subcortical tissue classes [37], [38], [39]. Freesurfer processing for volumetric T1-weighted images included: motion correction, brain extraction and removal of non-brain tissue using a hybrid watershed/surface deformation procedure [40]; automated spatial transformation and WM segmentation of subcortical volumetric structures [41]; intensity normalization, tessellation of GM/WM boundary and automated topology correction [42]; and surface deformation following intensity gradients to optimally place GM/WM and GM/CSF borders at the location where the greatest shift in intensity defines the transition to the other tissue class [37]. Image outputs from each stage of Freesurfer processing were visually inspected and edited by an experienced imaging analyst (S.M.).

Quantitative estimates were derived in a large set of spatially distinct region of interests (ROIs) that covered the whole brain [38]. Surface area and cortical thickness were estimated for cortical areas, and GM volume was estimated for subcortical ROIs. We also measured total intracranial volume (ICV). All ROI measures were normalized to a Z-score before evaluating between-group differences using a general linear model (MANCOVA) with ICV as a covariate and group status (WS versus TD) as the independent factor. The significance threshold was adjusted using the Benjamin-Hochberg false discovery rate (FDR; q<0.05) correction for multiple comparisons. Exploratory analyses were conducted to test for correlations between regional brain variations and Beck Anxiety Inventory scores using Pearson correlation statistics. Anxiety scores were not available for TD controls; therefore, correlation tests were only conducted within the WS group. Analyses were limited to brain regions that showed significant between-group differences. Due to the exploratory nature of the correlation analysis, results are reported at an unadjusted p-value<0.05.

## Results

**Figure 1:**
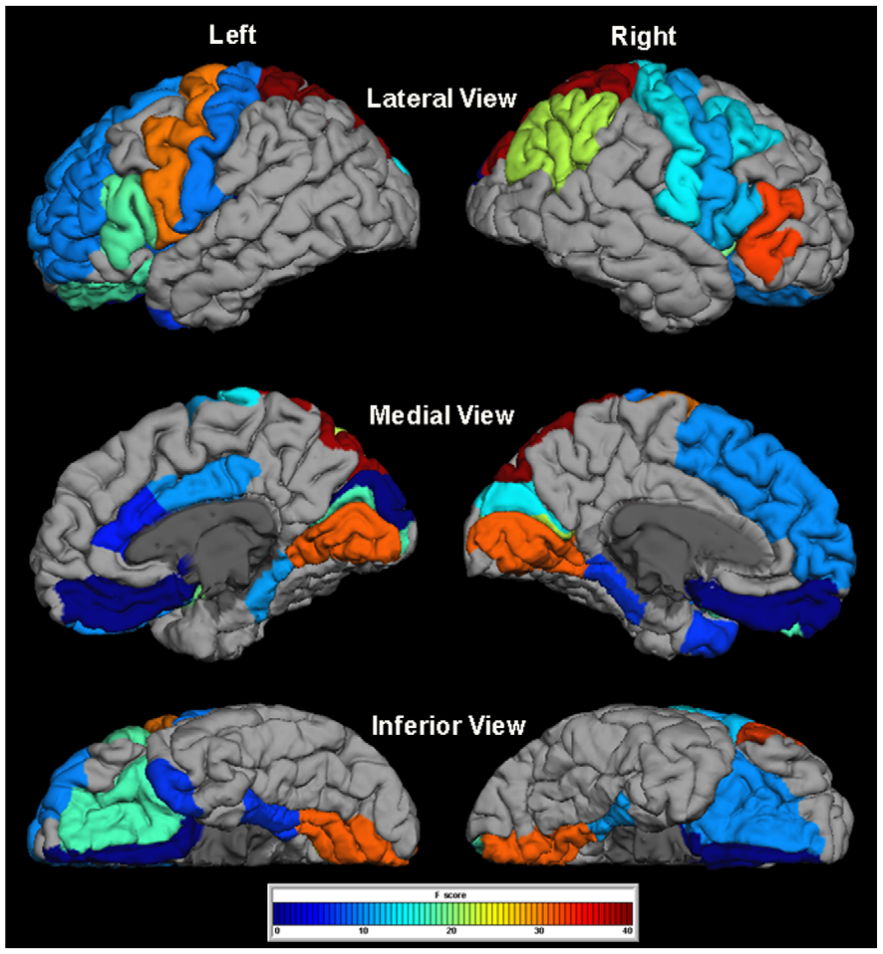
Significant reductions in gray matter surface area measures in WS versus TD controls. Figure 1 shows an overlay of F-test statistics (with values indicated by the color bar) on each Freesurfer-labeled ROI that was significantly different between groups. Surface area measures in gray regions were not significantly different between WS and TD.

**Figure 2:**
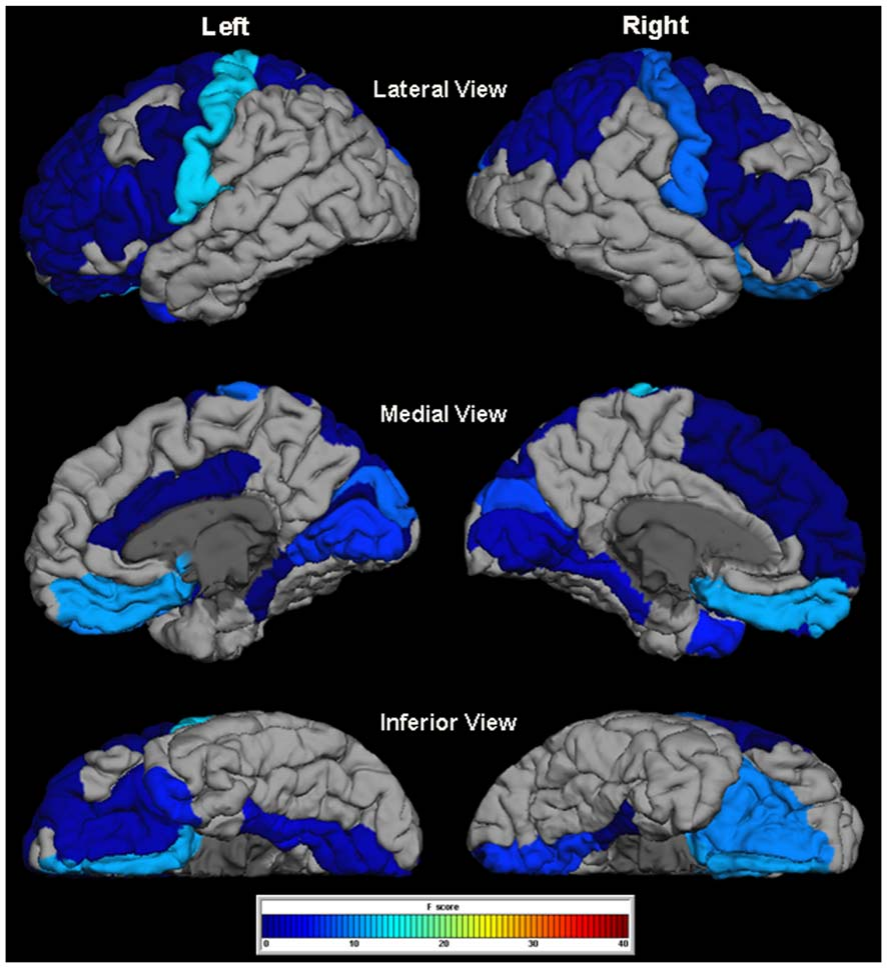
Significant increases in gray matter thickness measures in WS versus TD controls. Figure 2 shows an overlay of F-test statistics (with values indicated by the color bar) on each Freesurfer-labeled ROI that was significantly different between groups. Thickness measures in gray regions were not significantly different between WS and TD.

**Figure 3:**
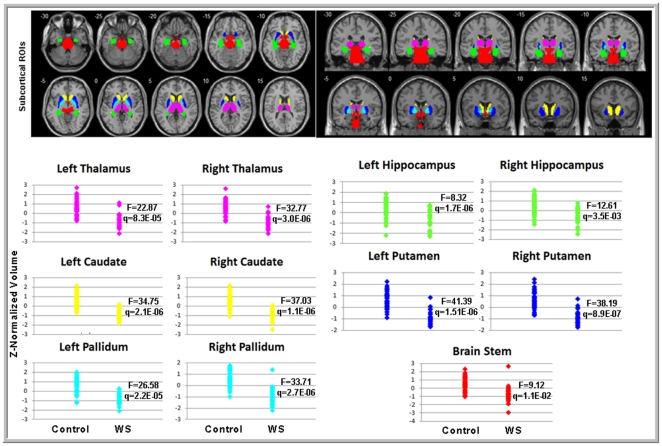
(Top) Color coded anatomical ROI masks of subcortical regions, which showed significant group differences in volume. (Bottom) Scatter plots of volume estimates (Z-normalized) in corresponding subcortical regions demonstrating overall decreases in volume in the areas in the WS brain versus TD controls. Presented statistical values correspond to F scores and FDR corrected q values derived from the MANCOVA. Colors in scatter plots correspond to in the color-coded anatomical ROIs in the Top portion of Figure 3.

There were no significant group differences in age or sex (see Table 1). For all cortical regions showing significant group differences in morphology, we observed a consistent pattern of lower surface area (SA) and greater cortical thickness (CT), although in some cases, only one measure reached significance for a given ROI. The cortical regions with significantly lower SA and significantly greater CT in the WS group compared to the TD control group included the left cuneus, left lateral OFC, right lingual gyrus, and bilateral postcentral gyrus. Only one region—the left temporal pole—showed significantly higher SA and significantly lower CT in WS versus TD controls. Additional regions were significantly different only for SA (21 ROIs lower in WS) or only for CT (4 ROIs greater in WS). Table 2 presents the test statistics and p-values for all regions showing a significant group difference in morphology at either SA or CT, along with annotations of morphological results from prior studies for these same ROIs. Figures 1 and 2 depict significant between group differences in cortical surface area and cortical thickness ROI measurements, respectively, overlaid on a standard reference brain. Figure 3 shows subcortical volume differences in the form of scatter plots.

**Table 2:** Complete list of cortical ROIs with significant group differences in surface area and/or thickness.

		Current Study						Prior Studies		
		Surface Area			Thickness			[11], [12], [14], [15]	[16]	[17]
Cortical ROI	Hemi-sphere	F	FDR q value	Higher(+) or Lower(−) in WS	F	FDR q value	Higher(+) or Lower(−) in WS	Volume/Concen-tration	Gyrifi-cation	Sulcal Depth
Caudal Anterior Cingulate	R	6.38	**4.05E-02**	**−**	0.21	NS	+		+	+
Caudal Middle Frontal Gyrus	R	14.49	**1.70E-03**	**−**	0.98	NS	+			
Cuneus	L	15.08	**1.41E-03**	**−**	7.84	**2.07E-02**	**+**	−[15]	+	−
	R	0.21	NS	−	9.14	**1.16E-02**	**+**		+	−
Inferior Parietal Cortex	R	22.41	**9.34E-05**	**−**	1.98	NS	+			+
Insular Cortex	L	9.95	**8.14E-03**	**−**	0.27	NS	+	+[11], [15], −[15]		−
	R	20.17	**2.21E-04**	**−**	1.65	NS	+			−
Lateral Orbital Frontal Cortex	L	18.02	**4.46E-04**	**−**	2.34	NS	+	+[11],−[14], [15]		
	R	11.1	**5.16E-03**	**−**	10.64	**6.25E-03**	**+**	+[11], −[14]		
Lingual Gyrus	L	31.85	**3.84E-06**	**−**	2.66	NS	+			
	R	31.6	**3.86E-06**	**−**	6.97	**3.03E-02**	**+**			
Medial Orbital Frontal Cortex	R	0.48	NS	−	11.9	**4.15E-03**	**+**			−
	L	0.43	NS	−	12.29	**3.78E-03**	**+**			−
Parahippocampal gyrus	R	11.82	**4.17E-03**	**−**	0.2	NS	+	−[15]		−
	L	7.33	**2.62E-02**	**−**	3.66	NS	+	−[15]		−
Pars Opercularis	R	13.19	**2.75E-03**	**−**	0.69	NS	+			+
	L	18.84	**3.64E-04**	**−**	2.44	NS	+			+
Pars Triangularis	R	32.92	**3.19E-06**	**−**	0.01	NS	+			
	L	18.65	**3.76E-04**	**−**	0.73	NS	+			
Pericalcarine Cortex	R	18.23	**4.26E-04**	**−**	0.17	NS	+			+
Postcentral Gyrus	L	10.09	**7.77E-03**	**−**	13.61	**2.34E-03**	**+**	−[11], +[15]	+	−
	R	15.07	**1.00E-03**	**−**	9.43	**1.02E-02**	**+**	−[11], [15], +[15]	+	
Posterior Cingulate Cortex	R	11.45	**4.84E-03**	**−**	0.73	NS	+		+	+
Precentral Gyrus	L	30.59	**5.17E-06**	**−**	0.1	NS	+		+	
	R	12.1	**3.87E-03**	**−**	0.44	NS	+		+	
Rostral Middle Frontal Gyrus	L	10.52	**6.49E-03**	**−**	1.05	NS	+			
Superior Frontal Gyrus	L	11.12	**5.38E-03**	**−**	0.27	NS	+	−[15]		
Superior Parietal Cortex	L	40.33	**1.08E-06**	**−**	1.67	NS	+	−[11], [12], [14], [15]		−
	R	39.53	**7.08E-07**	**−**	1.07	NS	+	−[11], [15]		−
Temporal Pole	L	7.21	**2.74E-02**	**+**	6.16	**4.37E-02**	**−**			
Transverse Temporal Gyrus	L	0.01	NS	−	12.76	**3.23E-03**	**+**			−

Exploratory correlation analyses identified several brain ROIs correlated with anxiety scores, such that higher anxiety scores were associated with lower regional surface area or thickness. In the WS group, surface area was negatively correlated with Beck Anxiety Inventory scores in the left and right insula, left precentral gyrus, left rostral middle frontal gyrus, left superior temporal lobe, left superior frontal lobe, right lateral OFC and right lingual gyrus, such that higher anxiety scores were related to lower surface area. In contrast, cortical thickness was positively correlated with anxiety scores in the postcentral gyrus, such that higher anxiety scores were associated with greater cortical thickness. It is important to note that while these correlation trends are interesting and have good face validity, none would have survived correction for multiple comparisons and, thus, should be interpreted with caution. Table 3 details all nominally significant correlations.

**Table 3:** Results from exploratory correlation analyses of brain morphological measures and Beck Anxiety Inventory scores in WS subjects.

				Beck Anxiety Index	
ROI	Hemi-sphere	Measure	N	Pearson Correlation Coefficient (r)	Unadjusted p-value
Insular Cortex	L	SA	30	−0.39	**0.03**
Parahippocampal Gyrus	L	SA	30	−0.21	NS
Pars Opercularis	L	SA	30	−0.45	**0.01**
Postcentral Gyrus	L	T	30	0.46	**0.01**
Precentral Gyrus	L	SA	30	−0.56	**0.001**
Rostral Middle Frontal Gyrus	L	SA	30	−0.36	**0.05**
Superior Frontal Gyrus	L	SA	30	−0.39	**0.04**
Superior Temporal Gyrus	L	SA	30	−0.39	**0.04**
Lingual Gyrus	R	SA	30	−0.38	**0.04**
Insula Cortex	R	SA	30	−0.37	**0.04**
Lateral Orbital Frontal Cortex	R	SA	30	−0.42	**0.02**
Posterior-cingulate Cortex	R	SA	30	−0.09	NS

## Discussion

Using simultaneous ROI measures of cortical surface area, cortical thickness and subcortical volume, we were able to investigate complex patterns in brain morphological differences in WS relative to TD control subjects. Further, we explored brain-behavior relationships to identify possible neural correlates of anxiety in this genetically-driven disorder.

One of the most striking phenotypic features of WS is the characteristic hypersociability, paired with heightened empathy and a lack of fear of strangers. However, this pattern of over-friendliness is also accompanied by increased non-social anxiety [3]. An fMRI study by Meyer-Lindenberg et al. [32] examined the neural correlates of social cognition abnormalities in WS and found a deficient negative feedback circuit between OFC and amygdala that might underlie non-social anxiety and social disinhibition in WS. Avery et al. [21] found structural connectivity differences between amygdala and OFC that support the anxiety-related phenotype of WS. Morphometric findings in the OFC for WS have been inconsistent, with some reports of increases [14] and other reports of decreases in OFC volume in WS versus controls [15]. We found increased cortical thickness in the right lateral OFC and bilateral medial OFC, paired with reduced surface area bilaterally in the lateral (but not medial) OFC, in WS versus TD. These data may lend support to a previously published meta-analysis of fMRI and lesion studies suggesting a medio-lateral OFC distinction, wherein the medial OFC was related to monitoring reward value of reinforcers and lateral OFC function was related to evaluating negative reinforcers of ongoing behavior [43]. We found relative preservation of amygdala volume in WS. Our finding is consistent with a previous report in WS that used a tensor-based morphometric technique [9]; but conflicts with another that used voxel-based morphometry to detect a significant increase in GM density and volume in the amygdala [15]. The above discrepancy could be due to a couple reasons: 1) the nature of the analytic method that we employed uses a surface based registration algorithm to do inter-subject registration that yields a superior matching of homologous cortical regions compared to volumetric techniques such as voxel-based morphometry and 2) the VBM study had a small sample size and very different demographic characteristics compared to ours. Consistent with the report by Cohen et al. (2010) [44] of decreased volume in insular cortex, our study found bilaterally decreased insular surface area (with no significant differences in thickness). Insular function is thought to be critical to processing introceptive awareness, motor control and social and emotional processing, all of which are affected in WS [3], [45], [46], [47], [48], [49], [50]. Taken together, our findings indicate that a pattern of complex structural variations in the OFC, amygdala and insula may underlie impaired social and emotional processing in WS.

Persons with WS often demonstrate an impaired ability to suppress inappropriate social behavior, coupled with hypersociability. Recently, Mobbs et al. [5] conducted a functional MRI experiment using the Go/NoGo task, concluding that individuals with WS failed to activate a frontal network of regions, including both cortical and sub-cortical regions such as the basal ganglia that are critical to successful behavioral inhibition. Reductions in basal ganglia and brain stem volume in WS have been consistently reported in previous studies using voxel- and tensor-based morphometry [9], [10]. Similarly, in the WS group from our study, we found a marked volumetric reduction in the brainstem and several subcortical regions within the basal ganglia. We also found evidence for decreased surface area (with no alterations in thickness) in other frontal regions such as the rostral middle frontal gyrus, caudal middle frontal gyrus, superior frontal gyrus and precentral gyrus. These findings lend further support for significant structural alterations in the fronto-basal circuit that might underlie impaired social restraint, inhibitory control of action and social cognition in WS [51], [52], [53].

Individuals with WS have difficulty performing tasks that require visual-spatial information to be analyzed and transmitted to motor and executive control processes primarily performed in the frontal lobes, such as drawing and block construction tasks, suggesting an overall deficiency in the visuospatial cognitive system governed by fronto-parietal circuits [54]. Reductions in surface area in the cuneus, inferior parietal, superior parietal, inferior frontal, medial frontal, superior frontal, premotor and lingual gyri seen in our study all lend further support to the widely accepted dorsal-visual stream impairment that likely underlies poor visuospatial abilities in WS [16], [54].

Even though short-term verbal working memory is a relative strength in WS [55], our study found reduced volume of the hippocampus and reduced surface area of the parahippocampal gyrus in WS. This might be consistent with the study results from Meyer-Lindenberg et al. [32] that used multi-modal imaging (positron emission tomography and fMRI) to show a profound reduction in blood flow to the hippocampal formation in WS.

We also found increased thickness and relatively preserved surface area of the transverse temporal gyrus in WS that might underpin an increased affinity to music, ability to remember lyrics and auditory sensitivity [4]. This is consistent with a report from Thompson et al. [56] that employed a different 3D cortical reconstruction method (using cortical pattern matching and fractal dimension analysis) to capture only thickness profiles in WS. They also found a pattern of increased gyrification and cortical thickness in WS in the perisylvian region that encompassed the superior temporal and transverse temporal gyri.

Although preliminary, our supplementary analyses aimed to shed more light on the influential role of observed structural alterations on levels of anxiety in WS. Individuals with WS show high rates of symptoms of generalized and anticipatory anxiety, and more than 50% of the population meets DSM-IV criteria for specific (non-social) phobias [1], [50]. In our study, participants with WS demonstrated mild-to-moderate levels of anxiety as measured by the Beck Anxiety Inventory questionnaire. Functional neuroimaging studies have attributed regional impairments in the amygdala and OFC to heightened anxiety in WS [33], [34], [57]. Killgore et al. [58] recently demonstrated that functional activation in the middle rostral insula was associated with anxiety sensitivity, or the tendency to fear the thoughts, symptoms and social consequences associated with the experience of anxiety, in controls and in individuals with social phobia. Insula has also been implicated in specific animal phobia [59].

In addition to the aforementioned regions, individuals with generalized anxiety disorder exhibit reduced regional cerebral blood flow in the frontal and temporal lobes [60], [61]. Our study consistently implicates these regions in anxiety that is characteristic of WS by showing significant negative correlations between anxiety scores and surface area in the insula, lateral OFC, superior temporal gyrus, and superior and rostral middle frontal gyri. However, none of the thickness measures were correlated with anxiety scores.

A novel aspect of our study was the concurrent investigation of cortical surface area and thickness as opposed to volume or thickness measurements alone. This enabled us to capture specific patterns of morphological differences in critical regions such as the lateral and medial OFC, postcentral gyrus, temporal pole, lingual gyrus and cuneus that are often inconsistently reported in WS studies that examine only volumetric differences [8]. It is possible that increased surface area of a particular region might be associated with decreased thickness or vice-versa, negating between-group volumetric differences. Thus, studies limited to volume- or tensor-based measurement might not capture these complex structural variations or their associations with neuropsychological scores.

Despite its novel aspects and strengths, our study was limited by the fact that we did not investigate differences in white matter (WM) pathologies. Future studies incorporating concurrent WM measurements using tensor-based techniques, such as DTI, could further inform structural pathology in WS. We also did not directly test for laterality differences, which might provide additional insight to help describe unique morphological differences. Future studies could use an analytic approach similar to ours but also include other clinical populations, such as Down syndrome or autism, to evaluate comprehensive WS brain-behavior differences in the context of other neurodevelopmental disorders. Also, it would be necessary and interesting to explore and replicate our brain-behavior correlations in larger samples and in control subjects to validate and elucidate behavioral relationships with structural brain variations. Alternative neuropsychological measures, which might better capture aspects of non-social anxiety most relevant to WS should be considered as well.

In conclusion, this is the first study to examine a comprehensive set of surface- and volume-based ROIs in WS using Freesurfer methods. Our results were consistent with previous reports and also identified novel structural differences in regions related to impaired visuospatial construction, impulsivity, and altered social and emotional processing in WS. Furthermore, we were able to elucidate the complexity of structural gray matter differences in our WS cohort by measuring regional increases and decreases in the cortical surface area and thickness. Additionally, the study tested for associations of specific structural variations with levels of anxiety in WS, in an effort to elucidate their roles in the disorder. Overall, the study demonstrates the utility of concurrently measuring independent structural phenotypes to investigate the complex brain-behavior relationships in a neurodevelopmental disorder such as WS.

## References

[1] Dykens EM (2003). Anxiety, fears, and phobias in persons with Williams syndrome.. Dev Neuropsychol.

[2] Mervis CB, John AE (2010). Cognitive and behavioral characteristics of children with Williams syndrome: implications for intervention approaches.. Am J Med Genet C Semin Med Genet.

[3] Morris CA (2010). The behavioral phenotype of Williams syndrome: A recognizable pattern of neurodevelopment.. Am J Med Genet C Semin Med Genet.

[4] Dykens EM, Rosner BA, Ly T, Sagun J (2005). Music and anxiety in Williams syndrome: a harmonious or discordant relationship?. Am J Ment Retard.

[5] Mobbs D, Eckert MA, Mills D, Korenberg J, Bellugi U (2007). Frontostriatal dysfunction during response inhibition in Williams syndrome.. Biol Psychiatry.

[6] Tsai SW, Wu SK, Liou YM, Shu SG (2008). Early development in Williams syndrome.. Pediatr Int.

[7] Hocking DR, Rinehart NJ, McGinley JL, Moss SA, Bradshaw JL (2011). A kinematic analysis of visually-guided movement in Williams syndrome.. J Neurol Sci.

[8] Jackowski AP, Rando K, Maria de Araujo C, Del Cole CG, Silva I (2009). Brain abnormalities in Williams syndrome: a review of structural and functional magnetic resonance imaging findings.. Eur J Paediatr Neurol.

[9] Chiang MC, Reiss AL, Lee AD, Bellugi U, Galaburda AM (2007). 3D pattern of brain abnormalities in Williams syndrome visualized using tensor-based morphometry.. Neuroimage.

[10] Reiss AL, Eliez S, Schmitt JE, Straus E, Lai Z (2000). IV. Neuroanatomy of Williams syndrome: a high-resolution MRI study.. J Cogn Neurosci.

[11] Eckert MA, Tenforde A, Galaburda AM, Bellugi U, Korenberg JR (2006). To modulate or not to modulate: differing results in uniquely shaped Williams syndrome brains.. Neuroimage.

[12] Boddaert N, Mochel F, Meresse I, Seidenwurm D, Cachia A (2006). Parieto-occipital grey matter abnormalities in children with Williams syndrome.. Neuroimage.

[13] Eckert MA, Galaburda AM, Mills DL, Bellugi U, Korenberg JR (2006). The neurobiology of Williams syndrome: cascading influences of visual system impairment?. Cell Mol Life Sci.

[14] Meyer-Lindenberg A, Kohn P, Mervis CB, Kippenhan JS, Olsen RK (2004). Neural basis of genetically determined visuospatial construction deficit in Williams syndrome.. Neuron.

[15] Reiss AL, Eckert MA, Rose FE, Karchemskiy A, Kesler S (2004). An experiment of nature: brain anatomy parallels cognition and behavior in Williams syndrome.. J Neurosci.

[16] Gaser C, Luders E, Thompson PM, Lee AD, Dutton RA (2006). Increased local gyrification mapped in Williams syndrome.. Neuroimage.

[17] Van Essen DC, Dierker D, Snyder AZ, Raichle ME, Reiss AL (2006). Symmetry of cortical folding abnormalities in Williams syndrome revealed by surface-based analyses.. J Neurosci.

[18] Marenco S, Siuta MA, Kippenhan JS, Grodofsky S, Chang WL (2007). Genetic contributions to white matter architecture revealed by diffusion tensor imaging in Williams syndrome.. Proc Natl Acad Sci U S A.

[19] Hoeft F, Barnea-Goraly N, Haas BW, Golarai G, Ng D (2007). More is not always better: increased fractional anisotropy of superior longitudinal fasciculus associated with poor visuospatial abilities in Williams syndrome.. J Neurosci.

[20] Arlinghaus LR, Thornton-Wells TA, Dykens EM, Anderson AW (2011). Alterations in diffusion properties of white matter in Williams syndrome.. Magn Reson Imaging.

[21] Avery SN, Thornton-Wells TA, Anderson AW, Blackford JU (2011). White matter integrity deficits in prefrontal-amygdala pathways in Williams syndrome.. Neuroimage.

[22] Winkler AM, Kochunov P, Blangero J, Almasy L, Zilles K (2011). Cortical thickness or grey matter volume? The importance of selecting the phenotype for imaging genetics studies.. Neuroimage.

[23] Panizzon MS, Fennema-Notestine C, Eyler LT, Jernigan TL, Prom-Wormley E (2009). Distinct genetic influences on cortical surface area and cortical thickness.. Cereb Cortex.

[24] Paul BM, Stiles J, Passarotti A, Bavar N, Bellugi U (2002). Face and place processing in Williams syndrome: evidence for a dorsal-ventral dissociation.. Neuroreport.

[25] Doyle TF, Bellugi U, Korenberg JR, Graham J (2004). “Everybody in the world is my friend” hypersociability in young children with Williams syndrome.. Am J Med Genet A.

[26] Dykens EM, Rosner BA (1999). Refining behavioral phenotypes: personality-motivation in Williams and Prader-Willi syndromes.. Am J Ment Retard.

[27] Levitin DJ, Cole K, Chiles M, Lai Z, Lincoln A (2004). Characterizing the musical phenotype in individuals with Williams Syndrome.. Child Neuropsychol.

[28] Levitin DJ, Cole K, Lincoln A, Bellugi U (2005). Aversion, awareness, and attraction: investigating claims of hyperacusis in the Williams syndrome phenotype.. J Child Psychol Psychiatry.

[29] Levitin DJ, Menon V, Schmitt JE, Eliez S, White CD (2003). Neural correlates of auditory perception in Williams syndrome: an fMRI study.. Neuroimage.

[30] Thornton-Wells TA, Cannistraci CJ, Anderson AW, Kim CY, Eapen M (2010). Auditory attraction: activation of visual cortex by music and sound in Williams syndrome.. Am J Intellect Dev Disabil.

[31] Schlaug G, Jancke L, Huang Y, Steinmetz H (1995). In vivo evidence of structural brain asymmetry in musicians.. Science.

[32] Meyer-Lindenberg A, Hariri AR, Munoz KE, Mervis CB, Mattay VS (2005). Neural correlates of genetically abnormal social cognition in Williams syndrome.. Nat Neurosci.

[33] Munoz KE, Meyer-Lindenberg A, Hariri AR, Mervis CB, Mattay VS (2010). Abnormalities in neural processing of emotional stimuli in Williams syndrome vary according to social vs. non-social content.. Neuroimage.

[34] Thornton-Wells TA, Avery SN, Blackford JU (2011). Using novel control groups to dissect the amygdala's role in Williams Syndrome.. Dev Cogn Neurosci.

[35] Ding J, Chen H, Qiu C, Liao W, Warwick JM (2011). Disrupted functional connectivity in social anxiety disorder: a resting-state fMRI study.. Magn Reson Imaging.

[36] Pietrini F, Godini L, Lazzeretti L, Benni L, Pracucci C (2010). [Neuroimaging and neurobiology of social anxiety].. Riv Psichiatr.

[37] Dale AM, Fischl B, Sereno MI (1999). Cortical surface-based analysis. I. Segmentation and surface reconstruction.. Neuroimage.

[38] Desikan RS, Segonne F, Fischl B, Quinn BT, Dickerson BC (2006). An automated labeling system for subdividing the human cerebral cortex on MRI scans into gyral based regions of interest.. Neuroimage.

[39] Fischl B, Sereno MI, Dale AM (1999). Cortical surface-based analysis. II: Inflation, flattening, and a surface-based coordinate system.. Neuroimage.

[40] Segonne F, Dale AM, Busa E, Glessner M, Salat D (2004). A hybrid approach to the skull stripping problem in MRI.. Neuroimage.

[41] Fischl B, van der Kouwe A, Destrieux C, Halgren E, Segonne F (2004). Automatically parcellating the human cerebral cortex.. Cereb Cortex.

[42] Segonne F, Pacheco J, Fischl B (2007). Geometrically accurate topology-correction of cortical surfaces using nonseparating loops.. IEEE Trans Med Imaging.

[43] Kringelbach ML, Rolls ET (2004). The functional neuroanatomy of the human orbitofrontal cortex: evidence from neuroimaging and neuropsychology.. Prog Neurobiol.

[44] Cohen JD, Mock JR, Nichols T, Zadina J, Corey DM (2010). Morphometry of human insular cortex and insular volume reduction in Williams syndrome.. J Psychiatr Res.

[45] Phan KL, Wager T, Taylor SF, Liberzon I (2002). Functional neuroanatomy of emotion: a meta-analysis of emotion activation studies in PET and fMRI.. Neuroimage.

[46] Singer T (2006). The neuronal basis and ontogeny of empathy and mind reading: review of literature and implications for future research.. Neurosci Biobehav Rev.

[47] Craig AD (2009). How do you feel–now? The anterior insula and human awareness.. Nat Rev Neurosci.

[48] Fink GR, Frackowiak RS, Pietrzyk U, Passingham RE (1997). Multiple nonprimary motor areas in the human cortex.. J Neurophysiol.

[49] Perez-Garcia D, Granero R, Gallastegui F, Perez-Jurado LA, Brun-Gasca C (2011). Behavioral features of Williams Beuren syndrome compared to Fragile X syndrome and subjects with intellectual disability without defined etiology.. Res Dev Disabil.

[50] Udwin O, Yule W (1991). A cognitive and behavioural phenotype in Williams syndrome.. J Clin Exp Neuropsychol.

[51] Aron AR, Durston S, Eagle DM, Logan GD, Stinear CM (2007). Converging evidence for a fronto-basal-ganglia network for inhibitory control of action and cognition.. J Neurosci.

[52] Jahfari S, Waldorp L, van den Wildenberg WP, Scholte HS, Ridderinkhof KR (2011). Effective connectivity reveals important roles for both the hyperdirect (fronto-subthalamic) and the indirect (fronto-striatal-pallidal) fronto-basal ganglia pathways during response inhibition.. J Neurosci.

[53] Masterman DL, Cummings JL (1997). Frontal-subcortical circuits: the anatomic basis of executive, social and motivated behaviors.. J Psychopharmacol.

[54] Atkinson J, Braddick O, Anker S, Curran W, Andrew R (2003). Neurobiological models of visuospatial cognition in children with Williams syndrome: measures of dorsal-stream and frontal function.. Dev Neuropsychol.

[55] Jarrold C, Baddeley AD, Hewes AK (1999). Genetically dissociated components of working memory: evidence from Down's and Williams syndrome.. Neuropsychologia.

[56] Thompson PM, Lee AD, Dutton RA, Geaga JA, Hayashi KM (2005). Abnormal cortical complexity and thickness profiles mapped in Williams syndrome.. J Neurosci.

[57] Meyer-Lindenberg A, Mervis CB, Berman KF (2006). Neural mechanisms in Williams syndrome: a unique window to genetic influences on cognition and behaviour.. Nat Rev Neurosci.

[58] Killgore WD, Britton JC, Price LM, Gold AL, Deckersbach T (2011). Neural correlates of anxiety sensitivity during masked presentation of affective faces.. Depress Anxiety.

[59] Rosso IM, Makris N, Britton JC, Price LM, Gold AL (2010). Anxiety sensitivity correlates with two indices of right anterior insula structure in specific animal phobia.. Depress Anxiety.

[60] de Bellis MD, Keshavan MS, Shifflett H, Iyengar S, Dahl RE (2002). Superior temporal gyrus volumes in pediatric generalized anxiety disorder.. Biol Psychiatry.

[61] Zhao XH, Wang PJ, Li CB, Wang JH, Yang ZY (2006). Prefrontal and superior temporal lobe hyperactivity as a biological substrate of generalized anxiety disorders.. Nat Med J China.

