# Correction: Regional Brain Differences in Cortical Thickness, Surface Area and Subcortical Volume in Individuals with Williams Syndrome

**DOI:** 10.1371/annotation/e4ec835e-932f-4956-b9fd-7113eee67347

**Published:** 2012-05-31

**Authors:** Shashwath A. Meda, Jennifer R. Pryweller, Tricia A. Thornton-Wells

There was an error in the funding statement. The correct funding statement is: This research was supported by the following grants: National Institute of Health Roadmap for Medical Research, Grant T32 MH075883; National Institutes of Health/National Institute of Child Health and Development P30-HD15052 to the Vanderbilt Kennedy Center, National Center for Research Resources (Vanderbilt Clinical and Translational Science Award grant 1-UL1-RR024975); a Vanderbilt University Discovery Grant, and National Institute of Aging P30AG036445-01. The funders had no role in study design, data collection and analysis, decision to publish, or preparation of the manuscript.

